# Do scientific attitude and intelligence affect motivation towards STEM? Structural equation modelling

**DOI:** 10.3389/fpsyg.2024.1481229

**Published:** 2024-11-13

**Authors:** Zeynep Dere

**Affiliations:** Department of Child Development, Ege University, İzmir, Türkiye

**Keywords:** STEM, scientific attitude, multiple intelligence, motivation, female leader candidate

## Abstract

**Introduction:**

The aim of this study is to reveal the effect of scientific attitude and intelligence on STEM (science, technology, engineering and mathematics) motivation using Structural Equation Modelling (SEM). It is possible to say that there is a reciprocal relationship between attitudes towards science, intelligence and motivation towards STEM. Motivation is closely and positively related to STEM, scientific attitude, intelligence and organizational development. When female students are supported and motivated positively, it is possible for them to be successful in STEM. It is possible to say that female students are the female leadership candidates of the future. Female leader candidates can play a role in future organizational development. Female leader candidates who are supported and motivated by their environment can take their place in organizational development. There is a gap in the research literature on this subject in Türkiye. This study fills an important gap in terms of sampling, research method and data analysis.

**Method:**

In the study, personal information form, Attitude Towards Scientific Research Scale, Multiple Intelligences Self-Perception Scale and STEM Value-Expectation Rating Scale were applied to 159 female undergraduate students who are disadvantaged in STEM field and studying at Ege University Ödemiş Faculty of Health Sciences.

**Results:**

It is possible to say that most female students have positive attitudes towards scientific research. According to the research results, as the reluctance to help researchers and negative attitudes towards research increase, motivation towards STEM decreases. As positive attitudes towards research and researchers increase, motivation towards STEM increases. In addition, increases in verbal–linguistic intelligence, logical-mathematical intelligence, spatial intelligence, interpersonal intelligence, intrapersonal intelligence and natural intelligence lead to an increase in positive attitudes towards research and thus STEM motivation.

**Conclusion:**

When the value expectations of future female leadership candidates for the STEM field were evaluated, it was found that 81.13% of the students had a medium level of value expectation evaluation level for the STEM field. This situation suggests that female students are not positively motivated for STEM in the family, school and peer environment.

## 1 Introduction

Technology spreads rapidly in the 21st century. It is possible to evaluate the 21st century as the age of knowledge, information and space (UN, 2021; UNESCO, 2023). There are new developments every day in all fields of science (Shelley and Kiray, 2018). Technological advancements contribute to the economic development of countries (OECD, 2024). Various sectors and working areas have emerged thanks to economic and technological progress (Zohuri, 2020). The 21st-century education model focuses on cultivating productive, inquisitive, critical thinking, and problem solving students. As technology advances in all societies, students’ fundamental life skills become compatible with the age of space and information (Alpaydın and Demirli, 2022; Paechter et al., 2020).

STEM (science, technology, engineering, and mathematics) provides opportunities for students to address problems from an interdisciplinary perspective (Freeman et al., 2019; Kennedy and Odell, 2024). It is aimed to provide knowledge and skills with a holistic educational approach covering the entire education process from preschool to higher education (Titrek et al., 2023; Yan et al., 2024). Different disciplines are brought together with STEM education. It becomes possible to achieve an inclusive education that supports quality learning, the application of existing knowledge to daily life, the development of life skills and high-level critical thinking. The STEM approach is important in terms of enabling the transformation of theoretical knowledge in the fields of science, technology, engineering and mathematics into practice and products (Kennedy and Odell, 2024; OECD, 2024; Sheth, 2023).

STEM education aims at economic progress (Hu and Guo, 2021; Yan et al., 2024). Countries are adopting various approaches to address gender divides. However, while 68% of countries globally have policies to support STEM education, only half of these policies specifically support girls and women. The United States has set a goal to increase the number of STEM bachelor’s degrees by one million over a 10-year period (Young et al., 2018). In Kenya, the Ministry of Education has organized STEM boot camps in schools, aiming to encourage and empower girls to pursue STEM studies and careers. In Namibia, it has mandated Goal 4 of the National Science and Technology Innovation Policy. It has improved gender inequality in STEM and the participation of women in science education. It has established programs that support the participation of girls as leaders and decision-makers in science careers. Zambia’s Technical Education, Vocational and Entrepreneurship Training Authority has established a platform that offers free digital skill courses targeted, among others, at women. In Bangladesh, the Eighth Five Year Plan 2020–25 has a dedicated section on STEM education for girls. During its G20 presidency, India launched the Tech Equity platform to empower women with digital literacy skills (UNESCO, 2024). Türkiye is one of the countries with the lowest rate of STEM graduates.

STEM fields are frequently promoted due to the high labour-market demand for graduates (Bybee, 2010). However, these fields also show gender disparities, with women under-represented in many STEM disciplines due to societal norms and educational biases. Progress in encouraging more women to pursue STEM-related fields has been slow. Across OECD countries, the proportion of female new entrants choosing to study STEM fields increased by less than 1 percentage point between 2015 and 2022 (OECD, 2024). It can be said that there is a strong positive correlation between individuals’ inclination towards STEM and their motivation (Master et al., 2017).

### 1.1 Motivation and STEM

Literature suggests that positive correlations between motivation and STEM, scientific attitude, intelligence and organizational development. According to Bandura’s Social Cognitive Theory, an individual’s performance and internal processes are combined. The individual’s behaviour is influenced by his/her cognitive level and environment (Schunk and DiBenedetto, 2019). Bandura explained how individuals are motivated, how they can motivate others, their performance and behaviours. The theory states that people influence others and are influenced by others. According to Bandura, individual factors and the environment mutually influence the behaviour of the individual (Koutroubas and Galanakis, 2022). When the interactions are positive, the subsequent behaviour of the individual is positively affected. In order to regulate behaviour, the individual first observes his/her own performance. He compares his performance with his standards. When he/she evaluates it positively, he/she is motivated to repeat the behaviour. Social environment plays an important role in motivating the individual (Fryling et al., 2011). It can be said that female students are the female leader candidates of the future. Motivation is an important factor in the orientation of female students towards STEM education (Dönmez, 2020).

Maslow’s theory forms the basis of motivation theories. It leads the way in explaining motivation in organizational development (Şengöz, 2022). Female leadership candidates will take their place in organizational development in the future. Therefore, the motivation of female students is important.

Motivation is the mediator between supportive environmental environment and entrepreneurial intention. It focuses on organization management. Its main purpose is to facilitate effective functioning and growth in an organization (Anbarasu, 2011; Singh and Ramdeo, 2020). In organizational development, leaders who can lead, produce both attitudes and creative ideas, and collaborate across disciplines are an important need (Pedraza-Rodríguez et al., 2023).

In organizational development, women’s leadership in STEM fields is based on motivation. When female students are positively motivated in STEM by their families, peers and school environment, they can assume strong female leadership roles in the future. A positive and supportive environment will motivate female students to STEM. It will be possible for female students to show motivated female leader characteristics in organizational development. Additionally, there is a positive relationship between STEM and attitudes toward science.

### 1.2 Attitudes towards science and STEM

In this study, it was first hypothesized that positive attitudes towards science would positively predict STEM motivation. Science stands out as a distinct field in the STEM approach. Science involves conducting experiments, making discoveries through observations, and conducting ethical research by wondering about the causes of events while following scientific procedures (Da Silva, 2022). Science is a dynamic process that explains observable phenomena directly or indirectly. It shows continuity, encompassing logical thinking processes. They can be defined as universal truths that do not change from one person to another or from observation to observation (Erduran and Dagher, 2014).

Attitudes toward science refer to positive attitudes toward science and scientists. There is a positive relationship between an individual’s attitudes toward science and their belief that they will be successful in science. Attitudes toward science are related to an individual’s interest and motivation to learn science (Cheung, 2017; Elliniadou and Sofianopoulou, 2022).

When individuals are positively motivated in scientific subjects, they are more successful in science and mathematics. Chang and Cheng (2008), focused on approximately 1.044 students in Taiwan. As a result, it was found that students are more successful in science when they are positively supported in science. Chiu and Klassen (2010), studied 88.590 students who participated in the PISA of the OECD. Positive correlations were found between students growing up in a motivating, tolerant environment and their success in mathematics. According to Bronfenbrenner’s ecological systems theory, family, peers, school, teachers, culture, and developed policies shape the future of the individual. For example; families who take their children to science museums at an early age encourage their children to develop positive attitudes towards science subjects (Tong and An, 2024). It is important for individuals to have sufficient intelligence potential for scientific studies (Limeri et al., 2020).

### 1.3 Multiple intelligence and STEM

In this study, it was secondly hypothesized that multiple intelligence areas would positively predict STEM motivation. Gardner (1993) based his theory of multiple intelligences on eight types: verbal–linguistic, logical-mathematical, musical, bodily-kinesthetic, spatial, interpersonal, intrapersonal, and naturalist intelligence. It is possible to say that biological and cultural dimensions are at the basis of her theory. It argues that different types of learning occur in different regions of the brain. In addition to biological factors, the development of intelligence is associated with culture. It is suggested that the types of intelligence and behavioral patterns valued by cultures are more developed (Morgan, 2021). Gardner proposes four criteria for a characteristic to be considered intelligence: symbols, being valued by the culture, being instrumental in producing goods or services, and problem-solving abilities (Davis et al., 2011). As society, economic policies and culture place more value on one of the multiple intelligence sub-dimensions, it may cause the individual to turn to that field as a profession.

Knowing the intelligence areas of students can help them to be successful in science, technology, engineering and mathematics. Determining the academic tendencies of STEM students provides opportunities for them to be motivated. Cabuquin (2022) examined the performance of students receiving STEM education in multiple intelligence areas and specialty subjects. For this purpose, he focused on 94 male and 99 female, 193 STEM students in total. According to the results; students who are motivated by their parents and teachers are more willing to learn and succeed in the STEM field. For example; students who are positively motivated by their families and teachers to solve mathematical problems are more likely to be successful in mathematics (Saadati and Celis, 2023). In addition, there are studies in the literature focusing on gender differences in STEM, which is approached from different perspectives.

### 1.4 Gender and STEM

This study focused only on female students. Because it can be said that girls are more disadvantaged than boys in STEM. Identifying the disadvantages of female students is important for the well-educated female leadership candidates who will take their place in organizational development in the future.

It is possible to say that the academic performance of female students is related to the motivation of the environment in which they grow up (Chiu and Klassen, 2010). While the low participation of women in STEM fields is generally explained by women’s lack of interest and skills in these fields, statistics contradict this. A study covering 67 countries through an international database published in the journal Studies in Psychology in 2018 showed that in two out of every three countries, girls outperform or perform equally with boys in STEM fields. In almost every country, it has been recognized that girls are more likely to pursue higher education in STEM fields than boys enrolled in these fields (Stoet and Geary, 2020). It can be said that female students are disadvantaged in STEM fields due to reasons such as family attitude, educational opportunities, and sexist approach (Huang et al., 2022).

Being exposed to gender stereotypes in society from an early age causes girls to avoid STEM fields. The idea that boys will be more successful than girls in mathematics is a social gender stereotype. The concept of “Leaky Pipeline” in the literature is defined as the negative impact of family-teacher-peers, as well as the lack of a female model that students can choose as a mentor in their educational life, and the fact that girls do not choose STEM fields or give up after choosing them due to their self-perception (Jakobs, 2022).

Female students who have a supportive environment with their parents, teachers and peers will be highly motivated. This will positively affect their academic skills. Supportive peer relationships and teacher behaviours provide students with the opportunity to be motivated (Kruse et al., 2024; Xu et al., 2023; Woreta, 2024). A supportive environment positively affects an individual’s entrepreneurial behavior. Motivation is a mediator between the environmental environment that positively supports female students and entrepreneurial intention. An individual’s entrepreneurial characteristics depend on being motivated for success (Lin et al., 2024). Family ties are the strongest bonds that most individuals have. Regardless of the type of bond, family ties are deep. This bond affects an individual’s behavior and decisions. An individual’s family can be encouraging or restrictive. An individual is motivated by family factors when making decisions. An individual’s motivation plays a mediating role between family ties and reaching a decision (Chauhan et al., 2024). When female students are motivated by their families, peers and school environment in STEM fields, they can assume leadership roles in these fields.

On average, women are overrepresented in education, but they are underrepresented in some fields. Only 15% of women STEM as a career, compared to 41% of men. These percentages have not changed since 2015 (OECD, 2024). According to the GEM report, the proportion of women among STEM graduates is 35% and has remained stable over the last 10 years (UNESCO, 2024). As norms around gender roles change, women may feel relatively more empowered in traditionally male-dominated fields such as STEM. Policies need to evolve to address women’s place in STEM (OECD, 2024).

Studies have been conducted with disadvantaged groups in the STEM field (Ertl et al., 2017; Kaplan and Yılmaz, 2021; Mutlu-Çaykuş and Korkut-Owen, 2017). However, the focus has not been on female undergraduate students. There is no study in the literature in Türkiye focusing on disadvantaged female undergraduate students in the STEM field (Karslı-Baydere et al., 2021; Lai and Cheng, 2023; Özkurt and Yakın, 2020). No previous research has been found that aims to develop a structural equation model to determine the effect of female undergraduate students’ scientific attitudes and intelligence on their motivation towards STEM (STEM Girls, 2020). This research will fill the gap in the literature. It is possible to say that the current research is pioneering. There are strong correlations between female students’ attitudes towards science, intelligence and motivation towards STEM. This relationship can be considered a guiding factor for students’ scientific progress.

There are STEM studies developed for disadvantaged girls and women in Türkiye. Some of the studies encouraging women to become engineers can be listed as follows; My Madame Curie,^1^ Stem for Disadvantaged Students Especially Girls,^2^ Sting,^3^ Honey Bees Become Engineers,^4^ Stem: Engineers of the Future, Türkiye’s Engineer Girls,^5^ Aziz Sancar Stem Camps for Girls Project,^6^ Science and Technology Seminar for Girls,^7^ Girls in Science and Technology,^8^ Girls Meet Science, My Steam Network,^9^ Stem School Project for Girls.^10^

This research aims to indicate the impact of scientific attitudes and intelligence on motivation towards STEM (science, technology, engineering, and mathematics) using Structural Equation Modeling (SEM). SEM is a statistical method based on explaining the causal and relational correlations between latent variables and observed variables in theoretical models. It allows for simultaneous evaluation of multiple dependent and independent variables and includes error values in the analysis process. It has numerous application examples in various fields, particularly in social and behavioral sciences (Savalei and Bentler, 2010).

## 2 Materials and methods

### 2.1 Population

The study focused on female students perceived as disadvantaged in terms of STEM. The population of the study consisted of 159 female students enrolled in Ödemiş Faculty of Health Sciences at Ege University during the 2023–2024 academic year.

### 2.2 Sample

The study used purposeful sampling. Purposive sampling procedures are used in most research papers. Because they are found in any research paradigm and help in ensuring that quality sample is located without biases so as to increase the reliability and trustworthiness of the findings (Nyimbili and Nyimbili, 2024). It employed a personal information form, the Scale of Attitude Towards Scientific Research, the Multiple Intelligence Self-Perception Scale, and the STEM Value-Expectancy Assessment Scale. Correlation analysis was utilized to examine the correlation between the scale scores used in the study. To decide on appropriate correlation analysis, the normal distribution status of the scale scores was first examined. As the scale scores showed a normal distribution, the Pearson correlation analysis was used. The SEM analysis was employed to examine the intermediary model established in the study.

### 2.3 Study group

The study focused on female students who were perceived as disadvantaged in terms of STEM. A total of 159 female students registered at Ege University Odemis Faculty of Health were included in the study. The group selected for SEM has high representativeness (Jobst et al., 2023). At the end of the semester, when the final exams were over, the female students were administered a personal information form, the Attitude Scale Towards Scientific Research, the Multiple Intelligence Self-Perception Scale and the STEM Value-Expectation Rating Scale online via Google Form. The responses of the students who volunteered to participate in the study were transferred to the data set. Table 1 shows attributes of the participants.

**Table 1:** Attributes of participants.

Variable	Min.	Max.	$\bar{X}$
Age	18	29	20.57

Variable	Variable level	*n*	%
Grade	1st Grade	49	30.82
	2nd Grade	60	37.74
	3rd Grade	37	23.27
	4th Grade	13	8.17
Number of siblings	No siblings	8	5.03
	One sibling	3	1.89
	Two siblings	55	34.59
	Three siblings	50	31.45
	Four siblings and more	43	27.04
Mother’s education background	Illiterate	12	7.55
	Primary school	67	42.14
	Secondary school	27	16.98
	High school	36	22.64
	University or higher	17	10.69
Father’s education background	Primary school	47	29.56
	Secondary school	32	20.13
	High school	55	34.59
	University or higher	25	15.72
What is the accommodation unit where you spend most of your life?	Village	25	15.72
	Town	3	1.89
	District	55	34.59
	Province	76	47.80
Do you study your department willingly?	Yes	139	87.42
	No	20	12.58
Have you previously received STEM education?	Yes	6	3.77
	No	153	96.23
Have you previously participated in a study related to STEM education?	Yes	10	6.29
	No	149	93.71

*n: Number of individuals; %, Percentage; Min., Minimum value; Max., Maximum value; $\bar{\text{X}}$, Mean.*

According to Table 1, the ages of the participants in the study range from 18 to 29 years, with an average of 20.57 years. Of the participants (*n* = 49), 30.82% are first grade, 37.74% (*n* = 60) are second grade, 23.27% (*n* = 37) are third grade, and 8.18% (*n* = 13) are fourth grade students. Of them, 5.03% (*n* = 8) have no siblings, 1.89% (*n* = 3) have one sibling, 34.59% (*n* = 55) have two siblings, 31.45% (*n* = 50) have three siblings, and 27.04% (*n* = 43) have four or more siblings. Among them, 7.55% (*n* = 12) have illiterate mothers, 42.14% (*n* = 67) have mothers with primary education, 16.98% (*n* = 27) have mothers with middle school education, 22.64% (*n* = 36) have mothers with high school education, and 10.69% (*n* = 17) have mothers with undergraduate and higher education. Of the participants’ fathers, 29.56% (*n* = 47) have primary education, 20.13% (*n* = 32) have middle school education, 34.59% (*n* = 55) have high school education, and 15.72% (*n* = 25) have university and higher education. Considering the settlement units where the participants spend most of their lives, 15.72% (*n* = 25) of them spend most of their lives in villages, 1.89% (*n* = 3) in small towns, 34.59% (*n* = 55) in districts, and 47.80% (*n* = 76) in cities. Among participants, 87.42% (*n* = 139) stated that they study their department willingly. Of the participants, 96.23% (*n* = 153) stated that they had not received STEM education before and 93.71% (*n* = 149) stated that they had not participated in a study on STEM education before.

### 2.4 Data analysis

Prior to the study analysis, incorrect data entry and missing data situations in the dataset were examined using frequency analysis. As a result of the examination, there were no missing data in the dataset. The identified incorrect data entries were corrected. To determine the participation levels of the participants to scale scores, they were classified into classes using k-means clustering analysis and the classes obtained based on class averages were named as low-medium-high. Correlation analysis was utilized to examine the correlation between the study’s scale scores. To decide on appropriate correlation analysis, the normal distribution status of the scale scores was first examined. Skewness and kurtosis coefficients were examined for the assumption of normal distribution and the results are presented in Table 2. For all scale scores, kurtosis values fell between −1 and + 1, and for skewness values, except for the positive attitude towards researchers and interpersonal intelligence subscales, these values were between −1 and + 1. For the positive attitude towards researchers and interpersonal intelligence subscales, the skewness values were −1.169 and −1.067, respectively. Although this showed a slight deviation from the threshold range, considering the low kurtosis values and the small size of the sample, these subscales were also deemed to exhibit normal distribution. As the scale scores showed a normal distribution, the Pearson correlation analysis was used. The SEM analysis was employed to examine the intermediary model established in the study.

**Table 2:** Skewness and kurtosis values for the scale scores.

	*n*	Skewness		Kurtosis	
		Statistics	Std. error	Statistics	Std. error
Reluctance to help researchers	159	0.497	0.192	−0.115	0.383
Negative attitude towards research	159	0.65	0.192	0.041	0.383
Positive attitude towards research	159	−0.209	0.192	−0.231	0.383
Positive attitude towards researchers	159	−1.169	0.192	0.861	0.383
Verbal–linguistic intelligence subscale	159	−0.109	0.192	−0.044	0.383
Logical-mathematical intelligence subscale	159	0.183	0.192	−0.185	0.383
Musical intelligence subscale	159	0.117	0.192	−0.525	0.383
Bodily-kinesthetic intelligence subscale	159	0.037	0.192	−0.318	0.383
Spatial intelligence subscale	159	0.056	0.192	−0.331	0.383
Interpersonal intelligence subscale	159	−1.067	0.192	0.718	0.383
Intrapersonal intelligence subscale	159	−0.821	0.192	0.395	0.383
Naturalist intelligence subscale	159	−0.235	0.192	0.256	0.383
STEM value-expectancy assessment scale	159	0.471	0.192	−0.172	0.383

### 2.5 Measurement tools

The disadvantaged female undergraduate students were administered a personal information form, the Scale of Attitude Towards Scientific Research, the Multiple Intelligence Self-Perception Scale, and the STEM Value-Expectancy Assessment Scale. The scales used were selected because they cover current approaches and issues in education. The tools assess the skills emphasized in international education programs (OECD, 2024; UNESCO, 2024). The scales used in this study were selected because they are suitable for measurement and evaluation in education (Kitchen et al., 2019), valid, and reliable.

1. *Personal Information Form:* The form includes questions about students’ grade, age, number of siblings, parents’ education level, whether they study in the department willingly, where they spend most of their lives, whether they have received STEM education before, and whether they have participated in STEM-related studies.
2. *The Scale of Attitude Towards Scientific Research:* The Scale of Attitude Towards Scientific Research was developed by Korkmaz et al. (2011). It was submitted to expert opinion for content validity. To determine the validity of the scale, item discrimination powers and exploratory and confirmatory factor analyses were calculated. To determine its reliability, internal consistency and stability levels were calculated. As a result of the confirmatory factor analysis of The Scale of Attitude Towards Scientific Research, the goodness of fit values were found as [χ^2^ (401, N = 372) = 830.28, *p* < 0.001, RMSEA = 0.054, SRMR = 0.051, GFI = 0.90, AGFI = 0.85, CFI = 0.95, NNFI = 0.95, IFI = 0.94]. The study found that the Scale for Attitudes Toward Scientific Research, consisting of four factors and 30 items, is a valid and reliable tool that can be used to assess undergraduate students’ attitudes toward scientific research. The five-point Likert-type scale consists of 30 items that can be grouped under four factors. Each item in the factors is rated as follows: Strongly Disagree (1), Disagree (2), Undecided (3), Agree (4), Strongly Agree (5). The increase in scores obtained in reply to students’ responses to the five-point Likert-type scale indicates an increase in negative attitude for the first factor (Reluctance to help researchers) and second factor (negative attitude towards research), while it indicates an increase in positive attitude for the third (positive attitude towards research) and fourth (positive attitude towards researchers) factors.
3. *The Multiple Intelligence Self-Perception Scale:* It is a Likert-type scale developed by Yeşil and Korkmaz (2010). For its content validity, expert opinion was obtained. To determine its validity, factor analysis, item-total correlations, and item discrimination power were calculated. Based on the data obtained, the 143-item scale consisting of eight subscales was found to be a valid and reliable tool for determining undergraduate students’ individual intelligence profiles. Regarding the internal consistency coefficients for the subscales of the scale, Cronbach’s Alpha ranged from 0.785 to 0.926. Considering the overall reliability coefficient of the Multiple Intelligence Self-Perception Scale, Cronbach’s alpha was 0.957. Confirmatory factor analysis was not included in the study on the development of the Multiple Intelligence Self-Perception Scale. The results of the exploratory factor analysis for this scale show that the Logical-Mathematical Intelligence dimension subscale consists of two different factors and the explained variance rate is 55.59%. The Linguistic Intelligence self-perception subscale was divided into three different factors. It explained 48.682% of the total variance. The Musical Intelligence self-perception subscale is further divided into two different factors, which account for 53,813% of the total variance. The Bodily-Kinesthetic Intelligence self-perception subscale is further divided into two different factors, which account for 51.410% of the total variance. The Spatial Intelligence self-perception subscale is further divided into two different factors, which account for 54.622% of the total variance. The Interpersonal Intelligence self-perception subscale is further divided into three different factors, which explain 43.529% of the total variance. The Intrapersonal Intelligence Subscale is divided into three factors explaining 42.942% of the total variance. The Naturalistic Intelligence subscale is divided into two different factors, which account for 48.528% of the total variance. The items were rated as follows: Never (1), Rarely (2), Sometimes (3), Often (4), Always (5). The results obtained from the subscales can be graded as follows: 20–35: Very low level, 36–51: Low level, 52–67: Moderate level, 68–83: High level, 84–100: Very high level. The lowest obtainable score from the scale is 20 and the highest is 100.
4. *The STEM Value-Expectancy Assessment Scale:* This scale was developed by Appianing and Van Eck (2018) to determine undergraduate students’ motivation towards STEM. The validity and reliability studies of the Turkish version of the STEM Value-Expectancy Assessment Scale were conducted by Acıksoz et al. (2020). Confirmatory factor analysis was conducted to verify the validity of the scale administered to 196 science teacher candidates selected through purposeful sampling and Cronbach’s alpha internal consistency coefficients were calculated for reliability assessment. In the reliability analysis, Cronbach’s alpha internal consistency coefficients were calculated as 0.87 for the whole scale, 0.82 for the perceived value component, and 0.82 for achievement expectations in the STEM career component. The scales used in this study were chosen because they are valid and reliable in the field. According to the CFA results of the STEM Value-Expectation Rating Scale, a good fit for all index values (χ^2^/df = 2.1, RMSEA = 0.075, CFI = 0.97, GFI = 0.90, AGFI = 0.85, SRMR = 0.058, IFI = 0.97, NFI = 0.94, NNFI = 0.96) and confirmed that the data obtained from the Turkish version of the scale were compatible with the theoretical structure of the original instrument. Considering validity and reliability results together, the scale adapted to Turkish society is a highly valid and reliable measurement tool that can be administered to undergraduate students. The instrument that uses a five-point Likert scale consists of 14 items, including seven reversed items. With a two-factor structure, the first seven items of the instrument measure the perceived value of STEM fields, while the next seven items measure expectations of success in STEM professions. The lowest score obtainable from the measurement tool is 14, while the highest is 70.

To determine the reliability of the scores obtained from the scales used in the study, Cronbach’s alpha reliability coefficient was calculated. The results are presented in Table 3.

**Table 3:** The reliability of scale scores.

		Cronbach’s alpha
The scale of attitude towards scientific research	Reluctance to help researchers	0.89
	Negative attitude towards research	0.90
	Positive attitude towards research	0.92
	Positive attitude towards researchers	0.93
The multiple intelligence scale	Verbal–linguistic intelligence subscale	0.89
	Logical-mathematical intelligence subscale	0.94
	Musical intelligence subscale	0.93
	Bodily-kinesthetic intelligence subscale	0.91
	Spatial intelligence subscale	0.96
	Interpersonal intelligence subscale	0.97
	Intrapersonal intelligence subscale	0.97
	Naturalist intelligence subscale	0.96
STEM value-expectancy assessment scale		0.84

According to Table 3, the Cronbach’s alpha values range from 0.89 to 0.93 for the subscales of the Scale of Attitude Towards Scientific Research, from 0.89 to 0.97 for the subscales of the Multiple Intelligence Scale, while the Cronbach’s alpha value is 0.84 for the STEM Value-Expectation Assessment Scale. The Cronbach’s alpha value for each scale score is above 0.70, indicating that the scale scores are reliable (Taber, 2018).

## 3 Results

Clustering analysis was conducted for each subscale to classify attitude levels of female health sciences faculty students, considered disadvantaged in terms of STEM, towards scientific research. The results are presented in Table 4.

**Table 4:** Participants’ attitude levels towards scientific research.

		*n*	%	Min.	Max.	$\bar{X}$	Statistics
Reluctance to help researchers	Low	68	42.77	8.00	15.00	11.31	*F* (2.156) =340.432 *p* = 0.000
	Moderate	85	53.46	16.00	31.00	22.69	
	High	6	3.77	33.00	40.00	37.50	
Negative attitude towards research	Low	80	50.31	9.00	17.00	11.91	F (2.156) =334.834 *p* = 0.000
	Moderate	76	47.80	18.00	34.00	24.11	
	High	3	1.89	40.00	45.00	42.00	
Positive attitude towards research	Low	14	8.81	7.00	14.00	10.43	F (2.156) =313.601 *p* = 0.000
	Moderate	107	67.30	15.00	28.00	22.36	
	High	38	23.90	29.00	35.00	33.08	
Positive attitude towards researchers	Low	5	3.14	6.00	10.00	8.20	F (2.156) =383.149 *p* = 0.000
	Moderate	44	27.67	13.00	23.00	18.86	
	High	110	69.18	24.00	30.00	28.02	

*n, Number of individuals; %, Percentage; Min., Minimum value; Max., Maximum value; $\bar{\text{X}}$, Mean; sd, Standard deviation.*

Table 4 indicates that the results of the clustering analysis for all scale scores are statistically significant (*p* < 0.05), indicating that the classification is interpretable. According to the clustering analysis results for the subscale scores of reluctance to help researchers, 42.77% of the participants (*n* = 68) have a low level of reluctance to help researchers, 53.46% (*n* = 85) have a moderate level, and 3.77% (*n* = 6) have a high level. According to the clustering analysis results for the subscale scores of negative attitudes towards research, 50.31% of the participants (*n* = 80) have a low level of negative attitude towards research, 47.80% (*n* = 76) have a moderate level, and 1.89% (*n* = 3) have a high level. Regarding the positive attitude towards research subscale scores, the clustering analysis results indicate that 8.81% of the participants (*n* = 14) have a low level of positive attitude towards research, 67.30% (*n* = 107) have a moderate level, and 23.90% (*n* = 38) have a high level. Regarding the positive attitude towards researchers subscale scores, the clustering analysis results indicate that 3.14% of the participants (*n* = 5) have a low level of positive attitude towards researchers, 27.67% (*n* = 44) have a moderate level, and 69.18% (*n* = 110) have a high level. In general, the majority of female students enrolled in the health sciences faculty have low to moderate levels of reluctance to help researchers and negative attitudes towards research, while having moderate levels of positive attitudes towards research and high levels of positive attitudes towards researchers.

Clustering analysis was conducted for each subscale to determine intelligence levels in the multiple intelligence subscales of female health sciences faculty students, considered disadvantaged. The results are presented in Table 5.

**Table 5:** Multiple intelligence of the participants.

		*n*	%	Min.	Max.	$\bar{X}$	Statistics
Verbal–linguistic intelligence subscale	Low	6	3.77	19.00	31.00	26.67	F (2.156) =148.552 *p* = 0.000
	Moderate	118	74.21	32.00	56.00	45.79	
	High	35	22.01	57.00	69.00	61.86	
Logical-mathematical intelligence subscale	Low	14	8.81	21.00	42.00	37.35	F (2.156) =207.686 *p* = 0.000
	Moderate	117	73.58	44.00	84.00	64.44	
	High	28	17.61	85.00	105.00	95.32	
Musical intelligence subscale	Low	27	16.98	19.00	37.00	29.56	F (2.156) =242.280 *p* = 0.000
	Moderate	107	67.30	38.00	71.00	53.97	
	High	25	15.72	73.00	91.00	80.56	
Bodily-kinesthetic intelligence subscale	Low	9	5.66	14.00	27.00	21.89	F (2.156) =149.537 *p* = 0.000
	Moderate	120	75.47	28.00	55.00	42.47	
	High	30	18.87	56.00	70.00	62.80	
Spatial intelligence subscale	Low	28	17.61	16.00	31.00	23.11	F (2.156) =228.085 *p* = 0.000
	Moderate	113	71.07	32.00	64.00	47.65	
	High	18	11.32	65.00	80.00	73.39	
Interpersonal intelligence subscale	Low	6	3.77	16.00	28.00	18.00	F (2.156) =346.898 *p* = 0.000
	Moderate	68	42.77	33.00	64.00	52.18	
	High	85	53.46	65.00	80.00	74.60	
Intrapersonal intelligence subscale	Low	8	5.03	20.00	39.00	27.13	F (2.156) =264.500 *p* = 0.000
	Moderate	79	49.69	40.00	79.00	64.43	
	High	72	45.28	80.00	100.00	90.01	
Naturalist intelligence subscale	Low	10	6.29	22.00	44.00	31.20	F (2.156) =211.596 *p* = 0.000
	Moderate	118	74.21	46.00	88.00	70.01	
	High	31	19.50	90.00	110.00	101.03	

*n, Number of individuals; %, Percentage; Min., Minimum value; Max., Maximum value; $\bar{\text{X}}$, Mean; sd, Standard deviation.*

Table 5 indicates that the results of the clustering analysis for all scale scores are statistically significant (*p* < 0.05), indicating that the classification is interpretable. According to the clustering analysis results for the verbal–linguistic intelligence subscale scores, 3.77% of the participants (*n* = 6) have low verbal–linguistic intelligence level, 74.21% (*n* = 118) have a moderate level, and 22.01% (*n* = 35) have a high level. Regarding the logical-mathematical intelligence subscale scores, the clustering analysis results indicate that 8.81% of the participants (*n* = 14) have low logical-mathematical intelligence level, 73.58% (*n* = 117) have a moderate level, and 17.61% (*n* = 28) have a high level. The clustering analysis of the musical intelligence subscale scores reveal that 16.98% of the participants (*n* = 27) have low musical intelligence level, 67.30% (*n* = 107) have a moderate level, and 15.72% (*n* = 25) have a high level. Regarding the bodily-kinesthetic intelligence subscale scores, the clustering analysis indicates that 5.66% of the participants (*n* = 9) have low bodily-kinesthetic intelligence level, 75.47% (*n* = 120) have a moderate level, and 18.87% (*n* = 30) have a high level. For the spatial intelligence subscale scores, the clustering analysis results show that 17.61% of the participants (*n* = 28) have low spatial intelligence level, 71.07% (*n* = 113) have a moderate level, and 11.32% (*n* = 18) have a high level. Regarding the interpersonal intelligence subscale scores, the clustering analysis reveals that 3.77% of the participants (*n* = 6) have low interpersonal intelligence level, 42.77% (*n* = 68) have a moderate level, and 53.46% (*n* = 85) have a high level. The clustering analysis of intrapersonal intelligence subscale scores indicates that 5.03% of the participants (*n* = 8) have low intrapersonal intelligence level, 49.69% (*n* = 79) have a moderate level, and 45.28% (*n* = 72) have a high level. For the naturalist intelligence subscale scores, the clustering analysis results show that 6.29% of the participants (*n* = 10) have low naturalist intelligence level, 74.21% (*n* = 118) have a moderate level, and 19.50% (*n* = 31) have a high level. Overall, the majority of female students have moderate levels of verbal–linguistic, logical-mathematical, musical, bodily-kinesthetic, spatial, and naturalist intelligence, while their interpersonal and intrapersonal intelligence levels are moderate to high.

To determine the STEM value-expectancy assessment levels of female students, considered disadvantaged groups in terms of STEM, classification was performed through clustering analysis for scale scores. The results are presented in Table 6.

**Table 6:** The participants’ levels of STEM value-expectancy assessment scale.

		*n*	%	Min.	Max.	$\bar{X}$	Statistics
STEM value-expectancy assessment	Low	11	6.92	26.00	37.00	34.00	F (2.156) =114.722 *p* = 0.000
	Moderate	129	81.13	38.00	59.00	46.61	
	High	19	11.95	60.00	70.00	63.26	

*n, Number of individuals; %, Percentage; Min., Minimum value; Max., Maximum value; $\bar{\text{X}}$, Mean; sd, Standard deviation.*

Table 6 indicates that the results of the clustering analysis for scale scores are significant (*p* < 0.05), indicating that the classification is interpretable. According to the clustering analysis results for STEM Value-Expectancy Assessment Scale scores, 6.92% of the participants (*n* = 11) have a low level of STEM value-expectancy assessment, 81.13% (*n* = 129) have a moderate level, and 11.95% (*n* = 19) have a high level.

To examine the correlation between scores of the Scale of Attitude Towards Scientific Research, the Multiple Intelligence Scale, and the STEM Value-Expectancy Assessment Scale, the Pearson correlation analysis was conducted. The results are presented in Table 7.

**Table 7:** Correlations between scale scores.

	1	2	3	4	5	6	7	8	9	10	11	12	13
1. Reluctance to help researchers	1												
2. Negative attitude towards research	0.673^**^	1											
3. Positive attitude towards research	−0.356^**^	−0.276^**^	1										
4. Positive attitude towards researchers	−0.387^**^	−0.525^**^	0.414^**^	1									
5. Verbal–linguistic intelligence subscale	−0.334^**^	−0.212^**^	0.491^**^	0.512^**^	1								
6. Logical-mathematical intelligence subscale	−0.149	−0.003	0.375^**^	0.190*	0.480^**^	1							
7. Musical intelligence subscale	0.033	0.138	0.219^**^	−0.026	0.426^**^	0.470^**^	1						
8. Bodily-kinesthetic intelligence subscale	−0.045	0.016	0.168*	0.208^**^	0.432^**^	0.409^**^	0.443^**^	1					
9. Spatial intelligence subscale	−0.129	−0.145	0.342^**^	0.206^**^	0.399^**^	0.285^**^	0.344^**^	0.349^**^	1				
10. Interpersonal intelligence subscale	−0.363^**^	−0.461^**^	0.293^**^	0.525^**^	0.266^**^	0.169*	0.042	0.145	0.351^**^	1			
11. Intrapersonal intelligence subscale	−0.383^**^	−0.389^**^	0.352^**^	0.475^**^	0.497^**^	0.299^**^	0.136	0.274^**^	0.426^**^	0.732^**^	1		
12. Naturalist intelligence subscale	−0.194*	−0.166*	0.309^**^	0.215^**^	0.352^**^	0.268^**^	0.228^**^	0.161*	0.524^**^	0.454^**^	0.578^**^	1	
13. STEM value-expectancy assessment scale	−0.356^**^	−0.348^**^	0.446^**^	0.313^**^	0.324^**^	0.328^**^	0.142	0.211^**^	0.254^**^	0.299^**^	0.401^**^	0.354^**^	1

0.05; ^**^p < 0.01.

Correlation coefficients <0.20 are considered very weak, 0.20–0.39 is considered weak, 0.40–0.59 is considered moderate, 0.60–0.79 is considered strong and > 0.80 is considered very strong (Papageorgiou, 2022).

The study findings first examined whether female leadership candidates’ positive attitudes toward science positively affected their STEM motivation. It is then determined whether the multiple intelligence areas of female undergraduate students positively affect their STEM motivation. Table 7 shows that there is no significant correlation between the subscales of unwillingness to help researchers, negative attitudes towards research and logical-mathematical intelligence, musical intelligence, bodily-kinesthetic intelligence and spatial intelligence (*p* > 0.05). However, there are significant negative medium-level correlations between the subscales of unwillingness to help researchers and interpersonal intelligence, interpersonal intelligence (*r* = −0.363; *r* = −0.383) and a significant negative low-level correlation with naturalistic intelligence (*r* = −0.194) (*p* < 0.05). Positive attitude towards research has moderately significant positive correlations with verbal–linguistic intelligence, logical-mathematical intelligence, spatial intelligence, interpersonal intelligence and naturalistic intelligence subscales (*r* = 0.491; 0.375; 0.342; 0.352; 0.309, respectively) and slightly significant positive correlations with musical intelligence, bodily-kinesthetic and interpersonal intelligence subscales (*r* = 0.219; 0.168; 0.293, respectively) (*p* < 0.05). However, there is no significant correlation between the positive attitude towards researchers subscale and the musical intelligence subscale (*p* > 0.05). The positive attitude toward researchers subscale has significant positive moderate correlations with verbal–linguistic, interpersonal, and intrapersonal intelligence subscales (*r* = 0.512; 0.525; 0.475, respectively) and significant positive moderate correlations with logical-mathematical, bodily-kinesthetic, spatial, and naturalistic intelligence subscales (*r* = 0.190; 0.208; 0.206; 0.215, respectively) (*p* < 0.05). In addition, there are significant negative moderate correlations between the STEM Value-Expectation Evaluation Scale and the unwillingness to help researchers and negative attitudes toward researchers subscales (*r* = −0.356; *r* = −0.348, respectively) (*p* < 0.05). In addition, there are significant positive moderate correlations between STEM Value-Expectations Assessment Scale and positive attitudes towards researchers and its subscales (*r* = 0.446; *r* = 0.313, respectively) (*p* < 0.05). However, there is no significant correlation between STEM Value-Expectations Assessment Scale and musical intelligence subscale (*p* > 0.05). There are significant positive low correlations between STEM Value-Expectations Assessment Scale and bodily-kinesthetic, spatial and interpersonal intelligence subscales (*r* = 0.211, 0.254, 0.299, respectively) (*p* < 0.05). There are significant positive moderate correlations between the STEM Value-Expectation Assessment Scale and the verbal–linguistic, logical-mathematical, interpersonal and naturalistic intelligence subscales (*r* = 0.324; 0.328, 0.401, 0.354, respectively) (*p* < 0.05). In summary, there is no correlation between STEM values and expectations and musical intelligence. However, as verbal–linguistic, logical-mathematical, bodily-kinesthetic, spatial, interpersonal, interpersonal and naturalistic intelligence levels increase, STEM values and expectations also increase. In addition, as verbal–linguistic, interpersonal, interpersonal intelligence and naturalistic intelligence increase, reluctance to help researchers and negative attitudes towards research decrease, and positive attitudes towards research and researchers increase. As reluctance to help researchers and negative attitudes towards research increase, expectations towards STEM decrease. As positive attitudes towards research and researchers increase, expectations towards STEM also increase. According to the results, as the reluctance to help researchers and negative attitudes towards research increase, motivation towards STEM decreases. This finding shows that as the positive attitudes of female leadership candidates towards helping researchers and research increase, motivation towards STEM increases. In Table 7, the insignificant relationships presented regarding the mediating effect of attitude towards scientific research on the effect of multiple intelligences on STEM value expectation were removed from the model. The models examined within this scope are presented below.

**Figure 1:**
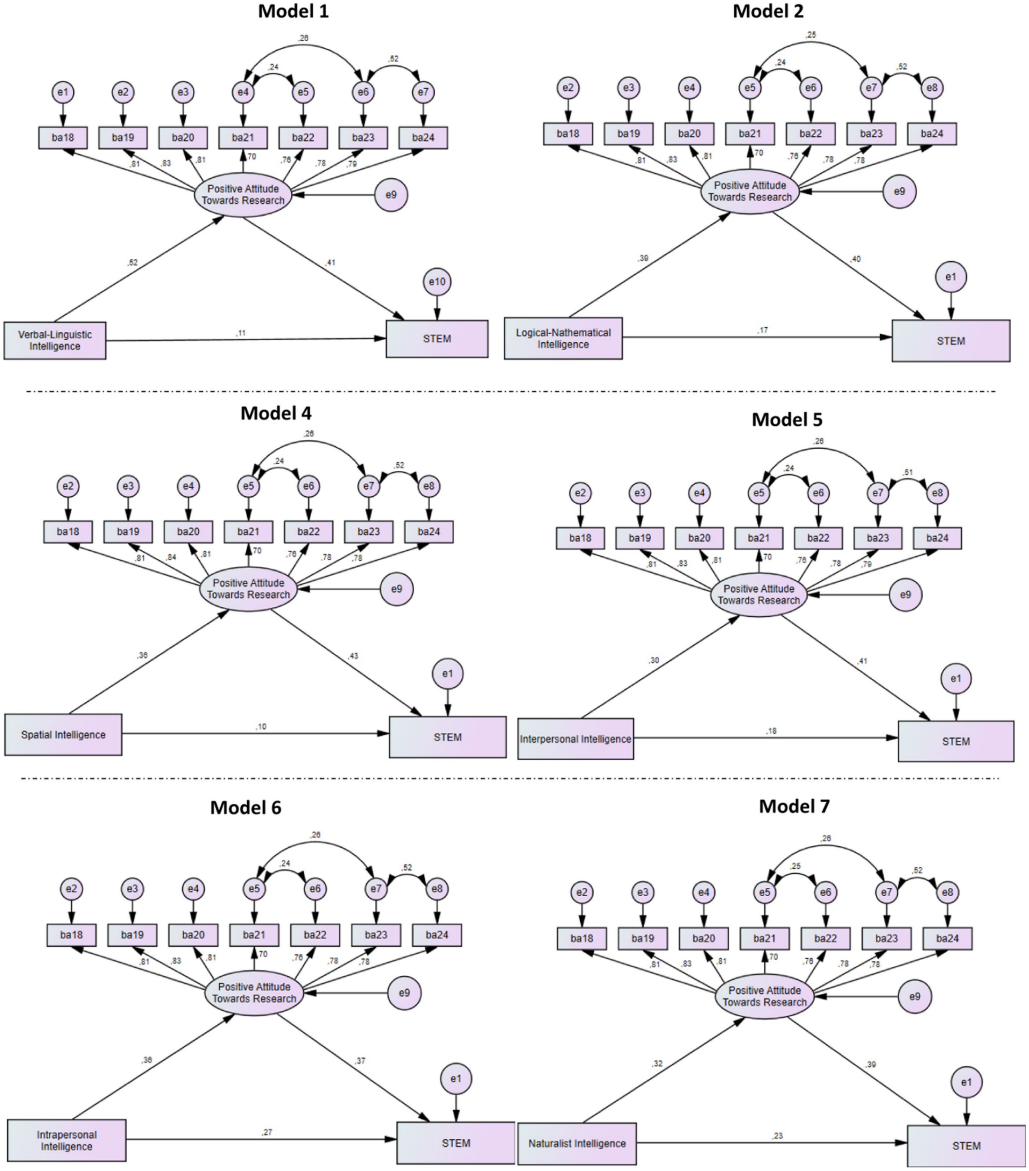
Path diagram of the effect of scientific attitude and intelligence on motivation towards STEM.

The results of the mediator variable analysis for the models shown in Table 8 are presented in Table 9. In addition, the path diagrams for the mediator models are given in Figure 1. Model 1 shows the mediating effects of unwillingness to help researchers, negative attitudes toward research, positive attitudes toward research, and positive attitudes toward researchers on the relationship between verbal–linguistic intelligence and STEM value expectation evaluations. When all four mediator variables are included in the model, the path coefficients from unwillingness to help researchers, negative attitudes toward research, and positive attitudes toward researchers to STEM value expectation evaluations are not significant (*p* > 0.05). Therefore, unwillingness to help researchers, negative attitudes toward research, and positive attitudes toward researchers do not mediate the correlation between verbal–linguistic intelligence and STEM value expectation evaluations. Therefore, these variables are removed from the model. As a result, only the variable positive attitudes toward research remains as the mediator variable. The value RMSEA = 0.109 for this model indicates that there is no model-data fit. The values of χ^2^/df = 2.890, GFI = 0.921, NFI = 0.928, CFI = 0.951 indicate that there is an acceptable model fit. In the analysis, when there is no mediator variable, the standardized path coefficient from verbal–linguistic intelligence scores to STEM value expectation assessment scale scores is 0.32 and is significant (*p* < 0.05). In Model 1, where positive attitudes towards the research variable are included as a mediator variable, the standardized path coefficient from verbal–linguistic intelligence scores to positive attitudes towards research scores (*β* = 0.52) and the standardized path coefficient from positive attitudes towards research scores to STEM value expectation assessment scale scores (*β* = 0.41) are significant (*p* < 0.05). When the mediator variable is included in the model, the standardized path coefficient from verbal–linguistic intelligence to STEM value expectation assessments is 0.11 and is not significant (*p* > 0.05). After the mediator variable is included in the model, the predictor variable does not have a significant effect on the predicted variable, so the mediator variable has a full mediation effect. Therefore, the effect of verbal–linguistic intelligence on STEM value expectation for female health sciences faculty students is fully mediated by positive attitudes towards research. In addition, the indirect effect of verbal–linguistic intelligence scores on STEM value expectation assessment scale scores through positive attitudes towards research is 0.21. In other words, as the verbal–linguistic intelligence level increases for female health sciences faculty students, it increases STEM value expectations through the effect of positive attitudes towards research.

**Table 8:** The intermediary variable models examined in the study.

	Predictor variable	Intermediary variable	Predicted variable
Model 1	Verbal–linguistic intelligence	Reluctance to help researchers	STEM value-expectancy assessment
		Negative attitude towards research	
		Positive attitude towards research	
		Positive attitude towards researchers	
Model 2	Logical-mathematical intelligence	Positive attitude towards research	STEM value-expectancy assessment
		Positive attitude towards researchers	
Model 3	Bodily-kinesthetic intelligence	Positive attitude towards research	STEM value-expectancy assessment
		Positive attitude towards researchers	
Model 4	Spatial intelligence	Positive attitude towards research	STEM value-expectancy assessment
		Positive attitude towards researchers	
Model 5	Interpersonal intelligence	Reluctance to help researchers	STEM value-expectancy assessment
		Negative attitude towards research	
		Positive attitude towards research	
		Positive attitude towards researchers	
Model 6	Intrapersonal intelligence	Reluctance to help researchers	STEM value-expectancy assessment
		Negative attitude towards research	
		Positive attitude towards research	
		Positive attitude towards researchers	
Model 7	Naturalist intelligence	Reluctance to help researchers	STEM value-expectancy assessment
		Negative attitude towards research	
		Positive attitude towards research	
		Positive attitude towards researchers	

**Table 9:** Intermediary variable results.

	Type	Effect	Estimate	*β*	z	*p*
Model 1	Indirect effect	Verbal–linguistic intelligence ⇒ AYOT ⇒ STEM	0.18	0.21		
	Component	Verbal–linguistic intelligence ⇒ AYOT	0.05	0.52	6.598	<0.05
		AYOT ⇒ STEM	3.76	0.41	4.498	<0.05
	Direct effect	Verbal–linguistic intelligence ⇒ STEM	0.10	0.11	1.347	0.178
	Total effect	Verbal–linguistic intelligence ⇒ STEM	0.28	0.32	4.302	<0.05
Model Fit indices: $\chi^{2}$/df = 2.890, GFI = 0.921, NFI = 0.928, CFI = 0.951, RMSEA = 0.109						
Model 2	Indirect effect	Logical-mathematical intelligence ⇒ AYOT ⇒ STEM	0.08	0.16		
	Component	Logical-mathematical intelligence ⇒ AYOT	0.02	0.39	4.816	<0.05
		AYOT ⇒ STEM	3.67	0.40	4.804	<0.05
	Direct	Logical-mathematical intelligence ⇒ STEM	0.08	0.17	2.259	<0.05
	Total	Logical-mathematical intelligence ⇒ STEM	0.16	0.33	4.363	<0.05
Model Fit indices: $\chi^{2}$/df = 3.051, GFI = 0.917, NFI = 0.922, CFI = 0.946, RMSEA = 0.114						
Model 4	Indirect effect	Spatial intelligence ⇒ AYOT ⇒ STEM	0.09	0.16		
	Component	Spatial intelligence ⇒ AYOT	0.02	0.36	4.495	<0.05
		AYOT ⇒ STEM	3.95	0.43	5.146	<0.05
	Direct effect	Spatial intelligence ⇒ STEM	0.05	0.10	1.275	0.202
	Total effect	Spatial intelligence ⇒ STEM	0.14	0.25	3.306	<0.05
Model Fit indices: $\chi^{2}$/df = 2.900, GFI = 0.920, NFI = 0.925, CFI = 0.949, RMSEA = 0.110						
Model 5	Indirect effect	Interpersonal intelligence ⇒ AYOT ⇒ STEM	0.07	0.12		
	Component	Interpersonal intelligence ⇒ AYOT	0.02	0.30	3.646	<0.05
		AYOT ⇒ STEM	3.80	0.42	5.510	<0.05
	Direct effect	Interpersonal intelligence ⇒ STEM	0.10	0.18	2.386	<0.05
	Total effect	Interpersonal intelligence ⇒ STEM	0.16	0.30	3.938	<0.05
Model Fit indices: $\chi^{2}$/df = 3.134, GFI = 0.913, NFI = 0.920, CFI = 0.943, RMSEA = 0.116						
Model 6	Indirect effect	Intrapersonal intelligence ⇒ AYOT ⇒ STEM	0.06	0.13		
	Component	Intrapersonal intelligence ⇒ AYOT	0.02	0.36	4.444	<0.05
		AYOT ⇒ STEM	3.39	0.37	4.636	<0.05
	Direct effect	Intrapersonal intelligence ⇒ STEM	0.12	0.27	3.645	<0.05
	Total effect	Intrapersonal intelligence ⇒ STEM	0.18	0.40	5.505	<0.05
Model Fit indices: $\chi^{2}$/df = 3.168, GFI = 0.913, NFI = 0.920, CFI = 0.943, RMSEA = 0.117						
Model 7	Indirect effect	Naturalist intelligence ⇒ AYOT ⇒ STEM	0.06	0.12		
	Component	Naturalist intelligence ⇒ AYOT	0.02	0.32	3.890	<0.05
		AYOT ⇒ STEM	3.61	0.39	4.946	<0.05
	Direct effect	Naturalist intelligence ⇒ STEM	0.10	0.23	3.135	0.005
	Total effect	Naturalist intelligence ⇒ STEM	0.16	0.35	4.758	<0.05
Model Fit indices: $\chi^{2}$/df = 2.826, GFI = 0.922, NFI = 0.927, CFI = 0.951, RMSEA = 0.107						

As seen in Table 8, Model 2 examines the mediating effect of positive attitudes toward research and positive attitudes toward researchers on the effect of logical-mathematical intelligence on STEM value expectation evaluations. When both mediating variables are included in the model, the path coefficients from positive attitudes toward researchers to STEM value expectation evaluations are not significant (*p* > 0.05). Therefore, positive attitudes toward researchers are removed from the model because there is no mediating effect on the effect of logical-mathematical intelligence on STEM value expectation evaluations. Finally, only positive attitudes toward the research variable remain as the mediating variable. As seen in Table 9, the model fit index for this model, RMSEA = 0.114, indicates that there is no model-data fit; while the values of χ^2^/df = 3.051, GFI = 0.917, NFI = 0.922, CFI = 0.946 indicate that there is an acceptable model fit. As a result of the analyses, the standardized path coefficient from logical-mathematical intelligence to value-expectation evaluations towards STEM is 0.33 and this effect is statistically significant (*p* < 0.05). In Model 2, where positive attitude towards research is included as a variable in the model, the standardized path coefficient from logical-mathematical intelligence dimension scores to positive attitude towards research dimension scores (*β* = 0.39) and the standardized path coefficient from positive attitude towards research dimension scores to value-expectation evaluations towards STEM scale scores (*β* = 0.40) are seen to be statistically significant (*p* < 0.05). When the mediator variable is included in the model, the standardized path coefficient from logical-mathematical intelligence to value-expectation evaluations towards STEM is 0.17 and is statistically significant (*p* < 0.05). The fact that the significant effect from the predictor variable to the predicted variable continues after the mediator variable is included in the model indicates that the mediator variable has a partial mediator effect. Accordingly, it can be said that the positive attitude towards the research variable has a partial mediating effect on the effect from logical-mathematical intelligence to STEM value expectation for women’s health sciences faculty students. In addition, the indirect effect from logical-mathematical intelligence dimension scores to STEM-focused value expectation assessment scale scores via positive attitude towards the research variable was determined as 0.16. In other words, the increase in the logical-mathematical intelligence level of women’s health sciences faculty students increases their value expectations towards STEM with the effect of positive attitude towards research. As seen in Table 8, Model 3 examines the mediating effect of positive attitudes towards research and positive attitudes towards researchers on the effect of bodily-kinesthetic intelligence on STEM value expectation evaluations. When both mediating variables are included in the model, the path coefficients from bodily-kinesthetic intelligence to positive attitudes towards research and from positive attitudes towards researchers to STEM value expectation evaluations are not significant (*p* > 0.05). This suggests that positive attitudes toward research or positive attitudes toward researchers do not have a mediating effect on the relationship between bodily-kinesthetic intelligence and STEM value expectancy assessments.

As seen in Table 8, Model 4 examines the mediating effect of positive attitudes towards research and positive attitudes towards researchers on the effect of spatial intelligence on STEM value expectation evaluations. When both mediation variables are included in the model, the path coefficients from positive attitudes towards researchers to STEM value expectation evaluations are not significant (*p* > 0.05). Therefore, positive attitudes towards researchers do not have a mediating effect on the effect of spatial intelligence on STEM value expectation evaluations and therefore it was removed from the model. As a result, only positive attitudes towards the research variable remained as a mediating variable. As seen in Table 9, the model fit index for this model, RMSEA = 0.110, indicates that there is no model-data fit; while the values of χ^2^/df = 2.900, GFI = 0.920, NFI = 0.925, CFI = 0.949 indicate that there is an acceptable model fit. As a result of the examination, it is seen that the standardized path coefficient from spatial intelligence to value-expectation evaluations towards STEM is 0.25 and this effect is statistically significant (*p* < 0.05). Positive attitude towards research is included as a variable in the model. In Model 2, the standardized path coefficient from spatial intelligence dimension scores to positive attitude towards research dimension scores (*β* = 0.36) and the standardized path coefficient from positive attitude towards research dimension scores to value-expectation evaluations scale scores for STEM (*β* = 0.43) are statistically significant (*p* < 0.05). When the mediator variable is included in the model, the standardized path coefficient from spatial intelligence to value-expectation evaluations towards STEM is 0.10 and is not statistically significant (*p* > 0.05). The fact that the significant effect from the predictor variable to the predicted variable does not continue after the inclusion of the mediator variable in the model shows that the mediator variable has a full mediator effect. Accordingly, it is seen that the positive attitude towards the research variable has a full mediation effect on the effect from spatial intelligence to the value-expectation towards STEM for female health sciences faculty students. In addition, the indirect effect from the spatial intelligence dimension scores to the value-expectation assessment scale scores for STEM through the positive attitude towards research variable was determined as 0.16. In other words, the increase in the spatial intelligence level of female health sciences faculty students increases their value-expectation towards STEM with the effect of the positive attitude towards research.

In Model 5, the mediating effect of unwillingness to help researchers, negative attitude towards research, positive attitude towards research and positive attitude towards researchers on the effect of interpersonal intelligence on value-expectation evaluations towards STEM is examined. When four mediator variables are included in the model, the path coefficients from the variables of unwillingness to help researchers, negative attitude towards research and positive attitude towards researchers to value-expectation evaluations towards STEM are not statistically significant (*p* > 0.05). In this case, the variables of unwillingness to help researchers, negative attitude towards research and positive attitude towards researchers do not have a mediating effect on the effect of interpersonal intelligence on value-expectation evaluations towards STEM and are removed from the model. In the last case, only the variable of positive attitude towards research is included as a mediator variable. As seen in Table 9, the value of RMSEA = 0.116 among the model fit indices for this model shows that there is no model data fit; χ^2^/df = 3.134, GFI = 0.913, NFI = 0.920, CFI = 0.943 values indicate that there is an acceptable model fit. As a result of the examination, the standardized path coefficient from interpersonal intelligence to value-expectation evaluations for STEM is 0.30 and this effect is statistically significant (*p* < 0.05). When positive attitude towards research is included in the model as a variable, the standardized path coefficient from interpersonal intelligence dimension scores to positive attitude towards research dimension scores in Model 5 is (*β* = 0.30), and the standardized path coefficient from research attitude dimension scores for STEM to value-expectation evaluation scale scores is (*β* = 0.42) and this effect is statistically significant (*p* < 0.05). When the mediator variable is included in the model, the standardized path coefficient from interpersonal intelligence to value-expectation evaluations for STEM is 0.18 and this effect is statistically significant (*p* < 0.05). After the mediator variable is included in the model, the significant effect from the predictor variable to the predicted variable continues, indicating that the mediator variable has a partial mediator effect. Accordingly, it can be said that the positive attitude towards the research variable has a partial mediator effect in the effect of interpersonal intelligence on the value-expectation towards STEM for female health sciences faculty students. In addition, it is determined that the indirect effect of the interpersonal intelligence dimension scores on the STEM-focused value-expectation assessment scale scores through the positive attitude towards the research variable is 0.12. In other words, the increase in the interpersonal intelligence level of female health sciences faculty students increases their value-expectations towards STEM with the effect of the positive attitude towards research.

In Model 6, the variables of unwillingness to help researchers, negative attitude towards research, positive attitude towards research and positive attitude towards researchers are found to mediate the effect of interpersonal intelligence on value-expectation evaluations towards STEM. When four mediator variables are included in the model, the path coefficients from the variables of unwillingness to help researchers, negative attitude towards research and positive attitude towards researchers to value-expectation evaluations towards STEM are not statistically significant (*p* > 0.05). In this case, the variables of unwillingness to help researchers, negative attitude towards research and positive attitude towards researchers do not mediate the effect of interpersonal intelligence on value-expectation evaluations towards STEM and are removed from the model. In the last case, only the variable of positive attitude towards research is included as a mediator variable. As seen in Table 9, the value of RMSEA = 0.117 among the model fit indices for this model indicates that the model data do not fit; χ^2^/df = 3.168, GFI = 0.913, NFI = 0.920, CFI = 0.943 values indicate acceptable model fit. As a result of the examination, the standardized path coefficient from personal intelligence to value-expectation evaluations towards STEM is 0.40 and this effect is statistically significant (*p* < 0.05). In Model 6, where positive attitude towards research is included as a variable in the model, the standardized path coefficient from personal intelligence dimension scores to positive attitude towards research dimension scores (*β* = 0.36) and the standardized path coefficient from research attitude dimension scores for STEM to value-expectation evaluations scale scores (*β* = 0.37) are statistically significant (*p* < 0.05). When the mediator variable is included in the model, the standardized path coefficient from personal intelligence to value-expectation evaluations towards STEM is 0.27 and is statistically significant (*p* < 0.05). The fact that the significant effect from the predictor variable to the predicted variable continues after the mediator variable is included in the model shows that the mediator variable has a partial mediator effect. Accordingly, it can be said that the positive attitude towards the research variable has a partial mediator effect on the effect coming from personal intelligence on the value expectation towards STEM for women health sciences faculty students. In addition, it is determined that the indirect effect from the personal intelligence dimension scores to the STEM-focused value expectation assessment scale scores through the positive attitude towards the research variable is 0.13. In other words, the increase in the personal intelligence level of women health sciences faculty students increases their value expectations towards STEM with the effect of the positive attitude towards research.

In Model 7, the mediating effects of the variables of unwillingness to help researchers, negative attitude towards research, positive attitude towards research and positive attitude towards researchers on the effect of natural intelligence on value-expectation evaluations towards STEM are analyzed. In the case of including four mediator variables in the model, the path coefficients from the variables of negative attitude towards diseases and positive attitude towards diseases to value-expectation evaluations towards STEM are significant (*p* > 0.05). In this case, the effect of helping with health improvements, negative attitude towards conditions and positive attitudes towards improvements on the effect of natural intelligence on value-expectation evaluations towards STEM is not specified and is removed from the model. In the last case, only the positive attitude variable is included as a mediator variable. Table 9 shows that there is no model-data fit with the model fit index RMSEA = 0.107 for modifying this model. The values of χ^2^/df = 2.826, GFI = 0.922, NFI = 0.927, CFI = 0.951 indicate an acceptable model fit. As a result of the examination, when there is no mediator variable, the standardized path from naturalistic intelligence to value-expectation evaluations towards STEM is 0.35 and this effect is consistently significant (*p* < 0.05). In Model 7, where positive attitudes towards research are included as model variance, the standardized paths from naturalistic breadth dimension scores to positive attitudes towards customers dimension scores (*β* = 0.32) and the standardized path from positive attitudes towards standards dimension scores to range scores of value-expectation evaluations towards STEM are consistently significant overall (*p* < 0.05) (*β* = 0.39). The change in the standardized path from naturalistic intelligence to value-expectation evaluations towards STEM is 0.23 and is permanently significant (*p* < 0.05). The fact that the significant effect from the predictor variable to the predicted variable continues after the inclusion of the mediator variable into the model shows that the mediator variable has a partial mediator effect. Accordingly, it can be said that the positive attitude towards research variable has a partial mediator effect in the effect of naturalistic intelligence on value-expectations towards STEM among female health sciences faculty students. In addition, the indirect effect of the positive attitude towards research variable from the naturalistic intelligence dimension scores to the value-expectation evaluation scale scores towards STEM is determined as 0.12. In other words, the increase in the intrapersonal intelligence levels of female health sciences faculty students increases their value-expectations towards STEM with the effect of the positive attitude towards research.

In conclusion, musical intelligence does not affect STEM values and expectations. An increase in bodily-kinesthetic intelligence enhances STEM values and expectations; however, no mediating effect was detected on attitudes towards scientific research. An increase in linguistic, logical-mathematical, spatial, interpersonal, intrapersonal, and naturalist intelligence increases STEM values and expectations, with only the positive attitude towards research acting as a mediating role in this relationship. In other words, high scores in verbal–linguistic intelligence, logical-mathematical intelligence, spatial intelligence, interpersonal intelligence, intrapersonal intelligence, and naturalistic intelligence lead to positive attitudes toward research. This positively affects female students’ STEM values and expectations.

## 4 Discussion

This study found that most female leadership candidates had low-moderate reluctance to help researchers and negative attitudes toward research, moderate positive attitudes toward research, and high positive attitudes toward researchers. It suggests that female leadership candidates who are likely to take part in organisational development in the future will exhibit high level positive attitudes towards research. Çelik et al. (2015) investigated nursing students’ anxiety levels and attitudes towards scientific research and various variables affecting them. As a result, it was found that the anxiety level of the students towards doing research was not very high and their attitudes towards doing research were positive. Björkström et al. (2003) investigated Swedish undergraduate nursing students’ attitudes towards and awareness of research and development in nursing. They focused on 201 students. As a result, they stated that nursing students’ attitudes towards research were positive. Kes and Öztürk-Şahin (2019) worked with nursing students. They worked with 162 students to determine students’ anxiety and attitudes towards scientific research. According to the results of the study, it was found that students’ attitudes towards research were positive. Korkmaz et al. (2011) developed an attitude scale to determine pre-service teachers’ attitudes towards scientific research. There were a total of 1,085 students in the study group. According to the results of the study, it was found that pre-service teachers’ attitudes towards research were positive. Ercoşkun (2019) examined pre-service teachers’ attitudes towards scientific research with various variables. In the study, 381 students were studied. Attitudes Towards Scientific Research Scale (ATSRS) was used as a data collection tool. According to the results of the study, it was found that pre-service teachers’ attitudes towards research were positive. Çakmak et al. (2015) examined pre-service social studies teachers’ attitudes towards scientific research. They worked with 259 students in the study in which the survey model was used. According to the results of the study, it was found that pre-service social studies teachers’ attitudes towards research were positive. Yılmaz et al. (2020) studied 57 undergraduate students to determine their attitudes towards scientific research. As a result, it was found that the students’ attitudes towards scientific research were positive. Halabi (2016) investigated the attitudes of nursing students in Saudi Arabia towards research. The study was conducted with 244 final year students in a three-campus nursing college in three regions of Saudi Arabia. As a result of the study using the survey model, it was found that the students’ attitudes towards scientific research were positive. Halabi and Hamdan-Monsour (2010) evaluated Jordanian nursing students’ attitudes towards nursing research. The study included 612 senior nursing undergraduate students studying in Oman, Jordan. As a result of the research using the survey model, it was determined that nursing students had positive attitudes towards research. Studies in the field overlap with the finding in this study. It was also found that different results were obtained in different studies. Polat (2014) aimed to examine the attitudes of education faculty students towards scientific research. In the research conducted with 417 students in total, a survey model was established. Attitudes Towards Scientific Research Scale (ATSRS) and a questionnaire were used as data collection tools. At the end of the study, it was seen that students’ attitudes towards scientific research were at a moderate level. Biçer et al. (2013) investigated the willingness of prospective Turkish teachers to scientific research. The study focused on 312 prospective teachers studying at two different state universities. At the end of the research, it was seen that the students’ attitudes towards scientific research were at a moderate level. A lack of self-belief in terms of mathematics and science aptitude limits girls’ and women’s science aspirations much more than their performance. A survey of more than 2,000 girls aged 15 to 19 in the Asia-Pacific region suggested that only 12% continued to study science subjects even though more than 50% were considering them when they were younger. It suggested that female students made these decisions due to gender discrimination, the difficulty of the courses, and their perception that they received inadequate social support from their families and teachers. Girls are more likely to experience math anxiety than boys. Mothers are also likely to be more anxious than fathers. They may be more likely to transmit math anxiety to their children, especially girls (UNESCO, 2024). These findings are consistent with the findings of this study. There are a number of challenges to women’s advancement in academic leadership. Some of these are unconscious biases and deep-rooted gendered expectations against women. Cultural beliefs about gender and gender biases that stem from workplace structures, practices, and interaction patterns that unintentionally favor men. Unconscious gender bias is a powerful but often invisible barrier to women’s advancement. Men have characteristics that are socially and anthropologically more adaptable to leadership roles, while women have poorer self-images, greater lack of confidence in women than men, and less career orientation and less interest in pursuing a career (Ayyildiz and Banoglu, 2024; Gamero, 2024; Marques et al., 2024).

It can be said that the verbal–linguistic, mathematical-logical, musical, bodily-kinesthetic, spatial-kinesthetic, spatial, naturalistic intelligence levels of most of the female students studying at the Faculty of Health Sciences are at medium level, while their interpersonal intelligence and intrapersonal intelligence levels are medium-high. Studies supporting the findings of these studies are frequently found in the literature. Mathematical ability and knowledge are critical for developing STEM skills and working in STEM fields. However, girls’ confidence in these subjects tends to be lower than boys’, even when they perform well. It is estimated that one in five people feel anxious about maths, but anxiety levels are higher among girls. In all educational systems participating in TIMSS in 2019, except Bahrain and Egypt, male students were found to be significantly more confident in mathematics than female students (UNESCO, 2024). One factor that has been shown to negatively affect mathematics performance and acquisition is mathematics anxiety. Math anxiety is generally more pronounced in women than in men. Gender stereotypes are believed to play a role in the high levels of mathematics anxiety (MA) reported by female students. Justicia-Galiano et al. (2023) examined gender stereotypes about mathematics anxiety (MA), its ability and affective components. It was investigated whether gender differences in MA were associated with gender stereotype beliefs in both ability and affective aspects. A total of 257 secondary school students completed measures of mathematics-related and gender stereotypes. It was examined whether there was a stereotypical belief that male and female adolescents experience mathematics anxiety to different degrees. The results clearly show that both gender groups believed that female students experience higher levels of mathematics anxiety than male students. The results regarding beliefs about mathematics anxiety are consistent with the pattern of gender differences found for beliefs about anxiety in general. Both male and female students believe that girls are more likely than boys to experience general anxiety-related emotions such as tension and worry. These findings suggest that negative emotions such as distress, sadness, fear, shame, and guilt in mathematics performance are experienced more by women. Regarding the emotional aspect of math anxiety experienced by both boys and girls, it was found that girls believed that they experienced math anxiety more than boys. The results showed that women tended to experience more negative emotions than men. It was concluded that gender differences in math anxiety may reflect actual levels of math anxiety rather than gender bias. Regarding the math ability stereotype, both girls and boys held egalitarian or pro-female views. It could be argued that being aware of the stereotype that boys will perform better than girls may have negative consequences for some girls, as the perception of these ideas may lead them to feel insecure and anxious in math situations. This finding shows that the traditional stereotype that positively discriminates against boys is gradually decreasing in Western societies. Girls who held the traditional math ability stereotype exhibited lower math self-confidence, lower math performance, and higher math anxiety. This finding highlights the relationship between gender, mathematical ability, self-confidence, beliefs about success, and mathematical anxiety. This suggests that female students who are likely to be future leaders should be more motivated to be successful in mathematical fields. Developing girls’ math skills is crucial to STEM. Van Mier et al. (2019) assessed the mathematics anxiety and mathematics performance of 124 s and fourth grade children, 67 girls and 57 boys. Although boys and girls showed roughly equal levels of mathematics anxiety and performed similarly on the arithmetic task, correlation analyses revealed that only in girls did mathematics anxiety correlate significantly with mathematics performance. Higher levels of math anxiety only significantly and negatively moderated math performance in girls. The results showed that math anxiety is already negatively linked to math performance in girls as early as second grade. Math anxiety has lifelong consequences on girls’ math performance, causing them to later avoid STEM careers. Preventing the development of math anxiety, especially in girls, from early childhood is essential. For girls who have encountered the negative consequences of math anxiety on math performance it is imperative to reduce the level of math anxiety. Therefore, intervention studies should be conducted to reduce girls’ mathematics anxiety. Gender-related effects of intervention programs implemented from an early age should be taken into account. İzci and Sucu (2014) examined the multiple intelligence profiles of university students. A total of 722 undergraduate students studying at a state university were included in the study. In the study, the intelligence areas of university students were evaluated with the “Multiple Intelligences Questionnaire.” Significant differences were found between the intelligence areas of university students in terms of gender variable. It was observed that female students had higher mean scores in visual–spatial and musical-rhythmic intelligence than male students. Pehlivan (2008) worked with 934 students to examine the differences between intelligence areas according to gender. The Multiple Intelligences Inventory was used to determine multiple intelligence areas. It was found that female students were more developed in verbal–linguistic, visual–spatial, musical-rhythmic and self-directed intelligence areas than male students. Loori (2005) examined the differences in the intelligence preferences of male and female students learning English as a second language in higher education institutions in the United States. In the study, it was found that female students’ interpersonal intelligence and intrapersonal intelligence levels were medium-high. İzci et al. (2007) examined the scores of the students enrolled in the dershanes in various intelligence areas in terms of their gender. A total of 387 students were included in the study and the Multiple Intelligences Scale was used. When the multiple intelligence areas of female students were examined, it was observed that they had high levels of linguistic, physical, musical, social and introverted intelligence areas. Altınok (2008) determined the multiple intelligence areas of students studying at the school of physical education and sports according to various variables. It was found that women’s musical-rhythmic intelligence levels were higher than men’s musical-rhythmic intelligence levels. In addition, it was determined that women’s visual–spatial intelligence and bodily-kinesthetic intelligence levels were higher than men’s visual–spatial intelligence and bodily-kinesthetic intelligence levels. These findings are similar to the findings of the study. Participating in physical exercises protects young people’s mental health and increases their motivation. Exercises can be said to improve mood and cognitive function. It reduces depressive symptoms, increases self-efficacy and improves emotional regulation (Li Z. et al., 2024). In addition, physical activities have positive effects on health motivation, appearance motivation, leisure motivation, ability motivation and social motivation of young people (Li J. et al., 2024). Teachers play a critical role in learning activities using multiple intelligence systems. It has been found that when teachers motivate their students to arts and sports, students’ motivation to study is also high. Making music has also been found to increase motivation (Immerz et al., 2024). This suggests that participation in physical and artistic activities would be beneficial for the motivation of female leadership candidates.

When the value expectations of future female leadership candidates for the STEM field were evaluated, it was found that 81.13% of the students had a medium level of value expectation evaluation level for the STEM field. This situation suggests that female students are not positively motivated for STEM in the family, school and peer environment. It is possible to say that males are less motivated in social sciences and females are less motivated in math-oriented fields such as physical sciences, technology, engineering and mathematics. It was determined that girls had a negative self-perception especially in mathematics. Therefore, it negatively affects girls’ motivation towards STEM. Female students negatively evaluate their skills, competencies and potential in mathematics. The negative motivation of female students causes them to move away from STEM fields in their career expectations and plans (Lazarides and Lauermann, 2019; Lazarides et al., 2016). Dietrich and Lazarides (2019) examined the development of motivational belief patterns in mathematics by gender over a school year and career plans in mathematics-related fields. They found that girls’ STEM motivation was not at a high level. This finding is in line with the research finding. This suggests that female leadership candidates who will take their place in organizational development need more motivation in STEM. The study showed that musical intelligence does not affect STEM values and expectations. A measurement tool measuring STEM values and expectations was used in this study. A measurement tool regarding STEAM motivation was not used. STEM is based on technical and cognitive skills such as science, technology, engineering and mathematics. STEM education programs focus only on the technical aspects of their disciplines. STEAM (STEM+Arts) is based on science, technology, engineering, mathematics and art-creativity. STEAM Education is a learning approach that uses Science, Technology, Engineering, Art and Mathematics as access points to guide students’ questioning, dialogue and critical thinking. Musical tendencies are related to art. The instrument measuring STEM motivation used in this study did not predict musical intelligence and art. In this case, it is thought that musical intelligence does not predict STEM motivation (Huang, 2020; Özer and Demirbatır, 2023; Turhal, 2020).

## 5 Recommendations

It is determined that most female leadership candidates have a low-medium level of reluctance to help researchers. It is seen that their positive attitudes towards research are medium and their positive attitudes towards researchers are high. Most of them have medium levels of verbal–linguistic, mathematical-logical, musical, bodily-kinesthetic, spatial and naturalistic intelligence. It is determined that their interpersonal intelligence and interpersonal intelligence levels are medium-high. 81.13% of female students in the Faculty of Health Sciences have a medium level of value-expectation evaluation level towards STEM. It can be said that it is possible to improve women’s skills towards STEM. Recommendations for parents, teachers, researchers, policy makers and female students are presented below for future women leaders to take a strong role in organisational development.

Suggestions for families;

- Girls should be positively motivated in STEM fields from an early age. Their mistakes should be tolerated and their successes should definitely be rewarded,
- Gender inequality in the family should be eliminated. The idea that “boys are better in mathematics and physics, girls are better at housework” should be abandoned,

Suggestions for teachers;

- Activities and opportunities that include teaching science, technology, engineering and mathematics together in an integrated manner in terms of application and concept can be offered to girls,
- Scientific study opportunities for girls in schools should be increased. Access to technology should be provided. The idea that “if you touch a computer, it will break, if it breaks, it cannot be repaired” should be abandoned,
- Educational programs that support mathematical-logical and spatial intelligence areas can be created in order to increase the motivation of female leadership candidates,
- Teachers should direct students to sports and art activities including music, physical-kinesthetic activities in order to increase the motivation of future female leaders.
- Female leadership candidates need to be motivated not only in social sciences but also in STEM,
- Gender norms in educational institutions should not be a disadvantage for female students. The prejudice that boys are better at solving problems and girls are not good should be eliminated,
- Future female leadership candidates need mentors who can act as positive role models. Therefore, female teachers should be able to act as role models,
- Various studies can be conducted to develop the leadership qualities of female students.

Suggestions for researchers.

- A “Leaky Pipeline” study can be conducted with women leaders in organizational development,
- This study was implemented only in the Faculty of Health Sciences. It is possible to implement it in other faculties at different scales,
- STEM is the subject of this study; STEAM may be the subject of another study.

Recommendations to policy makers;

- To promote non-discrimination and gender balance in technology and to motivate female leaders, investment should be made in programs that enable girls and young women to study and work in STEM fields,
- Work should be done to ensure female leadership in AI and technology development, gender-sensitive digital transformation, and address gender stereotypes in algorithms.

Recommendations for female leadership candidates;

- It is recommended that female leadership candidates participate in STEM education programs,
- Need to believe that they will be successful in STEM subjects and need to be able to motivate themselves to do so.

## Data Availability

The original contributions presented in the study are included in the article/supplementary material, further inquiries can be directed to the corresponding author.

## Footnotes

^1^https://ucansupurge.org.tr/benim-madame-curiem-projesi-2015-2016/ (Accessed September 5, 2024)

^2^https://www.aydin.edu.tr/tr-tr/arastirma/arastirmamerkezleri/cocuk/Pages/Projeler.aspx (Accessed September 5, 2024)

^3^https://stingeuproject.wordpress.com/ (Accessed September 5, 2024)

^4^https://www.rmkmarine.com.tr/bal-arilari-muhendis-oluyor-projesi.html (Accessed September 5, 2024)

^5^https://www.turkiyeninmuhendiskizlari.com/ (Accessed September 5, 2024)

^6^https://e-dergi.tubitak.gov.tr/edergi/yazi.pdf?dergiKodu=4&cilt=49&sayi=911&sayfa=6&yaziid=38892 (Accessed September 5, 2024)

^7^https://buyukcekmece.meb.gov.tr/www/kizlar-icin-stem-robotik-open-lab-projesi-egitimlerine-basvurular-basladi/icerik/813 (Accessed September 5, 2024)

^8^https://www.spacecampturkey.com/bilim-ve-teknolojide-kiz-cocuklari-uzayi-kesfetti (Accessed September 5, 2024)

^9^https://www.youtube.com/channel/UC_bGKm63kD4Y4ESeHYMahqw; https://www.youtube.com/@ucansupurgedernegi/featured (Accessed September 5, 2024)

^10^https://www.skdturkiye.org/esit-adimlar/guncel/kizlar-icin-stem-okulu- (Accessed September 5, 2024)
